# 

**Published:** 1892-05

**Authors:** 


					﻿Vol XII.
MAY, 1892.
NO. 5?
THE
pOMEOPATHlC pHY^lClAp,
A Monthly Journal of Homoeopathic Materia Medica
and Clinical Medicine.
"He is a freeman whom truth makes free, and all are slaves beside."
“ Seek the Truth: come whence it may, cost what it will."
EDITED AND PUBLISHED BY
WALTER M. JAMES, M. D.
PHILADELPHIA :
No. 1125 SPRUCE STREET.
LONDON:
ALFRED HEATH & CO., Sole Agents fob Great Britain,
114 Ebury Street, Eatou Square.
Yearly Subscription, $2.SO. invariably in advance. Single numbers, 96 cts
Entered at the Philadelphia Poet-Office ae Second-Class Mail Matter.
				

